# Characterization of Nanoparticle Dispersion in Red Blood Cell Suspension by the Lattice Boltzmann-Immersed Boundary Method

**DOI:** 10.3390/nano6020030

**Published:** 2016-02-05

**Authors:** Jifu Tan, Wesley Keller, Salman Sohrabi, Jie Yang, Yaling Liu

**Affiliations:** 1 Department of Mechanical Engineering and Mechanics, Lehigh University, Bethlehem, PA 18015, USA; jifutan@seas.upenn.edu (J.T.); sas713@lehigh.edu (S.S.); 2 Department of Civil and Environmental Engineering, Lehigh University, Bethlehem, PA 18015, USA; wjk207@lehigh.edu; 3 School of Mechanics and Engineering, Southwest Jiaotong University, Chengdu 610031, China; 4 Bioengineering Program, Lehigh University, Bethlehem, PA 18015, USA

**Keywords:** lattice Boltzmann method, immersed boundary method, cell suspension, nanoparticle delivery, dispersion rate

## Abstract

Nanodrug-carrier delivery in the blood stream is strongly influenced by nanoparticle (NP) dispersion. This paper presents a numerical study on NP transport and dispersion in red blood cell (RBC) suspensions under shear and channel flow conditions, utilizing an immersed boundary fluid-structure interaction model with a lattice Boltzmann fluid solver, an elastic cell membrane model and a particle motion model driven by both hydrodynamic loading and Brownian dynamics. The model can capture the multiphase features of the blood flow. Simulations were performed to obtain an empirical formula to predict NP dispersion rate for a range of shear rates and cell concentrations. NP dispersion rate predictions from the formula were then compared to observations from previous experimental and numerical studies. The proposed formula is shown to accurately predict the NP dispersion rate. The simulation results also confirm previous findings that the NP dispersion rate is strongly influenced by local disturbances in the flow due to RBC motion and deformation. The proposed formula provides an efficient method for estimating the NP dispersion rate in modeling NP transport in large-scale vascular networks without explicit RBC and NP models.

## 1. Introduction

Accurately predicting drug delivery is a critical task in drug development research and clinical trials [1,2]. It requires careful consideration of physiological conditions, such as hematocrit level (the volume ratio between red blood cell and the whole blood) [3,4], vessel geometry and flow conditions [5,6,7], drug carrier size and shape [3,8], dissolution rate [9] and external stimuli [10,11]. For small particles in red blood cell (RBC) suspensions, such as nanoparticles (NP) and platelets, recent studies have demonstrated that local flow field disturbances caused by RBC translation and deformation can enhance particle dispersion [3,12,13,14,15,16]. The migration of particles in RBC suspensions under shear has been shown to behave like a random walk process [17,18], with a dispersion rate much larger than thermal diffusion. Therefore, accurate predictions of NP dispersion in RBC suspensions must consider fluid-structure interaction between the immersed solid bodies (particles and cells) and the surrounding fluid. Previously-developed models for predicting NP dispersion in RBC suspensions have relied primarily on empirical data fitting. Aarts *et al.* experimentally studied shear-induced platelet diffusivity (*D*), which was fitted with shear rate (*η*) as a power law $D=k\eta^{n}$, where *k* is a constant and *n* is a function of hematocrit [19]. However, the model parameters are obtained empirically rather than predicted from the underlying physics. Decuzzi *et al.* extended the Taylor–Aris theory to calculate an effective NP diffusion rate that considers wall permeability and blood rheology [20,21]. They also reported about a three-fold increase in dispersion rate of 1 µm compared to thermal diffusion [16]. Recently, Fedosov’s group systematically studied micro- and nano-particles in drug delivery, including particle size, shape effect and RBC influence on particle margination and adhesion probabilities [3]. However, there is no analytical formula or quantitative rule to directly predict the NP dispersion rate so far, which is much needed in large-scale drug delivery simulations [20,21,22].

In order to address the deficiencies in previously-developed models for predicting NP dispersion, this paper presents a numerical study on NP dispersion in RBC suspensions that considers the effects of local flow field disturbances due to RBC motion. This study provides insight into the underlying physics driving NP dispersion in these systems and develops simple, yet effective, formulae for predicting dispersion rate as a function of characteristic physiological parameters. These simple predictive formulae will provide an efficient approach for assessing NP dispersion under different flow conditions and hematocrit level, thereby facilitating practical modeling of NP transport and distribution in large-scale vascular systems [22].

The remainder of this paper is organized as follows. The fluid-structure interaction model is introduced in Section 2, including a description of the modeling approach for the fluid environment, RBCs and NPs, as well as the fluid-structure coupling scheme. Section 3 outlines the model setup and test parameters for a parametric study on NP dispersion. Simulation results from the parametric study are presented in Section 4, along with formulae derived from a regression analysis for predicting the NP dispersion rate. Predictions from the formulae are compared to data reported in the literature. Finally, conclusions and recommendations from the study are presented in Section 5.

## 2. Fluid-Structure Interaction Model

The transport of particles in RBC suspensions is governed by hydrodynamic forces and fluid-structure interaction effects. In order to simulate this two-way coupling between the fluid environment and the immersed soft matter, a numerical fluid-structure interaction (FSI) code was developed utilizing an immersed boundary coupling scheme. Previous research has shown that the immersed boundary method (IBM) is an efficient approach to simulate soft matter and biological cells [23,24,25,26,27,28,29,30], flapping insect wings [31,32,33], harmonic oscillation of thin lamina in fluid [34] and other FSI problems, such as particle settling [35]. The advantage of this approach is two-fold. First, the lattice Boltzmann method (LBM) is very efficient at modeling fluid flow and ideal for parallel computing [36]. Second, the IBM uses an independent solid mesh moving on top of a fixed fluid mesh, thus removing the burden of re-meshing in conventional arbitrary Lagrangian–Eulerian methods [37]. In this study, the fluid was modeled using a lattice Boltzmann scheme, while the RBCs were modeled as elastic membranes. The fluids inside and outside the cell membrane are modeled with the same viscosity, following that in other works [38,39]. This approach greatly simplified the computation and without losing too much generality. It also can capture the multiphase feature of the blood flow. NPs were treated as rigid point elements with motions governed by both hydrodynamic loading and Brownian dynamics. Additional details regarding the fluid-structure interaction model are provided in the following sections.

### 2.1. Lattice Boltzmann Fluid Model

The lattice Boltzmann method (LBM) is a mesoscale approach to modeling fluid dynamics that has been used extensively in blood flow modeling [24,38,40]. Reviews of the underlying theory for the LBM can be found in the literature [41,42,43,44]. LBM is usually considered as a second order accurate method in space and time [45]. The key concept of the LBM is the transport of particles, characterized by a local density distribution function *f*_i_ (*x*,*t*) in phase space (*x*, ${\vec{c}}_{i}$), where *t* denotes the time and ${\vec{c}}_{i}$ denotes the lattice velocity. The dynamics of the LBM involves streaming and collision processes.

(1) $$\underset{\text{Streaming}}{\underset{︸}{f_{i}\left(x+\Delta t{\vec{c}}_{i},t+\Delta t\right)-f_{i}\left(x,t\right)}}=\underset{\text{Collision}}{\underset{︸}{{\;\Omega \;}_{i}}}+F_{i}$$

where $\Omega_{i}$ is the collision operator and $F_{i}$ is the external force term. The simplest and most computationally-efficient collision scheme is the Bhatnagar–Gross–Krook (BGK) scheme [46],

(2) $$\;\Omega_{i}=\frac{1}{\tau }(f_{i}\left(x,t\right)-f_{i}^{eq}\left(x,t\right))$$

where *τ* is a relaxation constant and $f_{i}^{eq}\left(x,t\right)$ is the population distribution at equilibrium, which is related to the local macroscale fluid velocity and the speed of sound $c_{s}$.

(3) $$f_{i}^{eq}\left(\vec{u},\rho \right)=w_{i}\rho \left(1+\frac{{\vec{c}}_{i}\cdot \vec{u}}{{c_{s}}^{2}}+\frac{{\left({\vec{c}}_{i}\cdot \vec{u}\right)}^{2}}{2{c_{s}}^{4}}-\frac{\vec{u}\cdot \vec{u}}{2{c_{s}}^{2}}\right)$$

where $w_{i}$ is the weight coefficients. An effective fluid viscosity is related to the relaxation time *τ* and the speed of sound *c_s_*,

(4) $$\;\nu =c_{s}^{2}\left(\tau -0.5\right)=\frac{\left(\tau -0.5\right)}{3}$$

and local macroscale density and velocity are calculated as:

(5) $$\rho \left(x,t\right)=\underset{i}{\sum }f_{i}(x,t),\rho \left(x,t\right)\vec{u}\left(x,t\right)=\sum_{i}f_{i}(x,t){\vec{c}}_{i}+\frac{\Delta t}{2}\vec{f}$$

Disturbances in the flow are introduced through a force density term *F_i_*, which can be expressed in terms of an external body force density $\vec{f}$ and fluid macroscale velocity $\vec{u}$,

(6) $$F_{i}=(1-\frac{1}{2\tau })w_{i}\left(\frac{{\vec{c}}_{i}-\vec{u}}{{c_{s}}^{2}}+\frac{{\vec{c}}_{i}\cdot \vec{u}}{{c_{s}}^{4}}{\vec{c}}_{i}\right)\cdot \vec{f}$$

For simplicity, the simulations presented in this paper consider a regular 2D domain with a D2Q9 (2D 9 velocities) fluid lattice, the details of which can be found in [45]. However, it is noted that the modeling approach is readily adaptable to the 3D case (see Figure S9 in the Supplementary Materials) and with more complex geometric configurations. Velocity and non-slip wall boundaries were used. There are several ways to implement velocity boundary conditions, such as Zou/He boundaries [47] and regularized boundary conditions [48]. The basic idea behind Zou/He boundaries is assuming that the bounce back rule applies to the non-equilibrium part of the density population. The regularized boundary condition is to use the moment flux tensor to reconstruct the non-equilibrium part of the density distribution, thus it is more stable, but requires extra calculation. Interested readers are referred to [49] for a summary of different boundary conditions in LBM. In this paper, treatment of velocity boundary conditions followed the recommendations in [47] and is discussed in Section 3.

### 2.2. Spring Connected Network Cell Membrane Model

**Figure 1:**
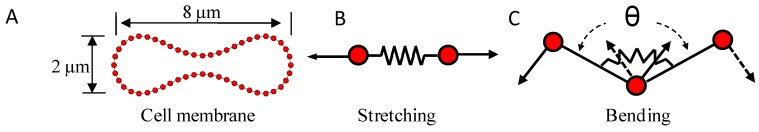
(**A**) Spring connected network cell membrane model. Kinematics for local stretching (**B**) and bending (**C**) modes of response.

Cell models are commonly based on a continuum approach utilizing a strain energy function to define membrane response [50,51,52]. Recently, however, a particle-based cell model governed by molecular dynamics has emerged as a popular alternative due to its simplicity in mathematical description [53,54,55]. These two models have been shown to provide consistent predictions [53]. For the present study, a particle-based RBC model is adopted. The model consists of a network of vertices, *X_i_*, $i\in \{1...N_{V}\}$, connected with elastic springs, which are effective in axial extension/contraction and flexure, as shown in Figure 1.

The potential energy of the membrane is defined as:

(7) $$V\{X_{i}\}=V_{stretch}+V_{bending}+V_{area}$$

The stretch energy mimics the elastic spectrin network and is given by:

(8) $$V_{stretch}=\frac{1}{2}k_{s}{\sum_{j\in 1...N_{s}}\left(L_{j}-L_{j0}\right)}^{2}$$

where *k_s_* is the stretching constant, $L_{j}$ is the length of the spring and $L_{j0}$ is the equilibrium spring length. To consider the nonlinear effect of membrane stretching, Fedosov investigated a wormlike chain potential [56]. In a separate study, Wida developed an exponential relationship for mechanical stiffness related to the bond stretch ratio (*λ*) [57]. In this paper, the same exponential form of the spring constant, $k_{s}=k_{s0}\exp\left[2(\lambda -1)\right]$, where λ is the bond stretch ratio, has been adopted.

The bending energy represents the bending resistance of the lipid bilayer and is defined as:

(9) $$V_{bending}=\frac{1}{2}k_{b}{\sum_{j\in 1...N_{b}}\left(\theta_{j}-\theta_{j0}\right)}^{2}$$

where $k_{b}$ is the bending constant and $\theta_{j}$ and *θ_j_*_0_ are instantaneous and equilibrium angles between adjacent spring, respectively. In this model, we used the angles when cells are at rest as the equilibrium angles *θ_j_*_0_; each angle is also different from the others.

When the cell deforms, the cell surface area and volume remain relatively constant. To maintain a constant area in the 2D model, an artificial restoring force potential $V_{area}=\frac{k_{a}{\left(A-A_{0}\right)}^{2}}{2A_{0}}$ is introduced to conserve the area where A is the instantaneous area, $A_{0}$ is the equilibrium area and $k_{a}$ is a constant.

The equilibrium shape of cells is followed by the analytical curve from Fung [58], as shown in Figure 1. The cell membrane was discretized to 52 nodes and angles. The equilibrium bond length was set to be equal, but the equilibrium angle varies among all of the angles.

### 2.3. Immersed Boundary Coupling Scheme

The immersed boundary method (IBM) was selected to model the interaction between the fluid and the immersed solids due to the algorithm’s efficiency. The IBM was first proposed by Peskin to study blood flow in the heart [59]. The fluid is solved on a spatially-fixed Eulerian grid, while the immersed solids are modeled using a moving Lagrangian mesh, which is not constrained to the geometric layout of the Eulerian fluid grid. Data are exchanged between the two domains through nodal interpolation. The coupling scheme enforces velocity continuity at the fluid-structure boundary and transfers forces from the structure back into the fluid through an effective force density. This two-way coupling automatically handles immersed body contact and prevents solid penetration through the development of restoring forces in the fluid. The approach has been used for a variety of fluid-structure interaction problems, including the simulation of jelly fish [60], blood flow [4,24,26,27,28,29,30,38] and platelet migration [14]. Comprehensive reviews of the IBM and its applications can be found in [25,61]. It is noticed that the current coupling approach works great for soft deformable solids, an alternative coupling approach, e.g., treating a solid as a moving boundary for the fluid and transferring the force back to the solid, should be used if the solid is relatively rigid, such as a particulate settling study [44,62,63,64].

The immersed structure can be viewed as a parametric surface **X**(*s,t*), where the force exerted by the structure on the fluid is interpolated as a source term in the momentum equation using:

(10) f(x,t)=∫F(s,t)δ(x−X(s,t))ds

where F(s,t) is the structural force at location *s* at time of *t* and *ds* is the discretized length of the immersed structure. The force is spread to the local fluid nodes through a delta function δ(r),

(11) $$\delta (r)=\{\begin{array}{c} \begin{array}{cc} \frac{1}{4}\left(1+\cos\left(\frac{\pi r}{2}\right)\right) & -2\leq r\leq 2 \end{array}\\ \begin{array}{cc} 0 & \text{ otherwise} \end{array} \end{array}$$

Other delta functions can also be used. Peskin listed a few delta functions in [25].

The structure velocity is interpreted from the local fluid field through:

(12) u(X,t)=∫u(x,t)δ(x−X(s,t))dx

The same delta function Equation (11) is used to interpolate cell node velocities from the local fluid flow field.

### 2.4. Nanoparticle Model

NPs were modeled as rigid bodies with motion governed by hydrodynamic forces and Brownian dynamics [65,66]. Langevin dynamics was used to simulate the motion of particles.

(13) $$m\frac{du}{dt}=-\varsigma u+F_{c}+F_{r}$$

where ς is the friction coefficient, $F_{c}$ is the conservative force and $F_{r}$ is the random force that satisfies the fluctuation dissipation theorem.

(14) $$\langle F_{r}(t)\rangle =0$$

(15) $$\langle F_{r}(t)F_{r}(t')\rangle =2\;k_{B}T\varsigma \;\delta (t-t')\;I$$

where *k_B_T* is the thermal energy, *δ(t − t′)* is the Dirac delta function and **I** is the unit-second order tensor.

The solution of the above equation gives $u(t)=\frac{F_{c}+F_{r}}{\varsigma }+C\exp(-\frac{\varsigma t}{m})$, where *C* is a constant. When the time step *dt* (4.2 × 10^−8^ s in the present study) is much larger than the relaxation time $\tau_{R}=\frac{m}{\varsigma }$(2.2 × 10^−9^ s), the particle position can be updated with the terminal velocity as $u(t)=\frac{F_{c}+F_{r}}{\varsigma }+u_{f}$. A position check was performed to avoid the penetration of particles into the RBCs, e.g., the random displacement induced by thermal fluctuation will be reversed if it will jump inside cells at the next time step. In the diffusion coefficient calculation, periodic boundary conditions were applied to particles when they crossed the channel wall to remove the effect of geometric confinement.

**Figure 2:**
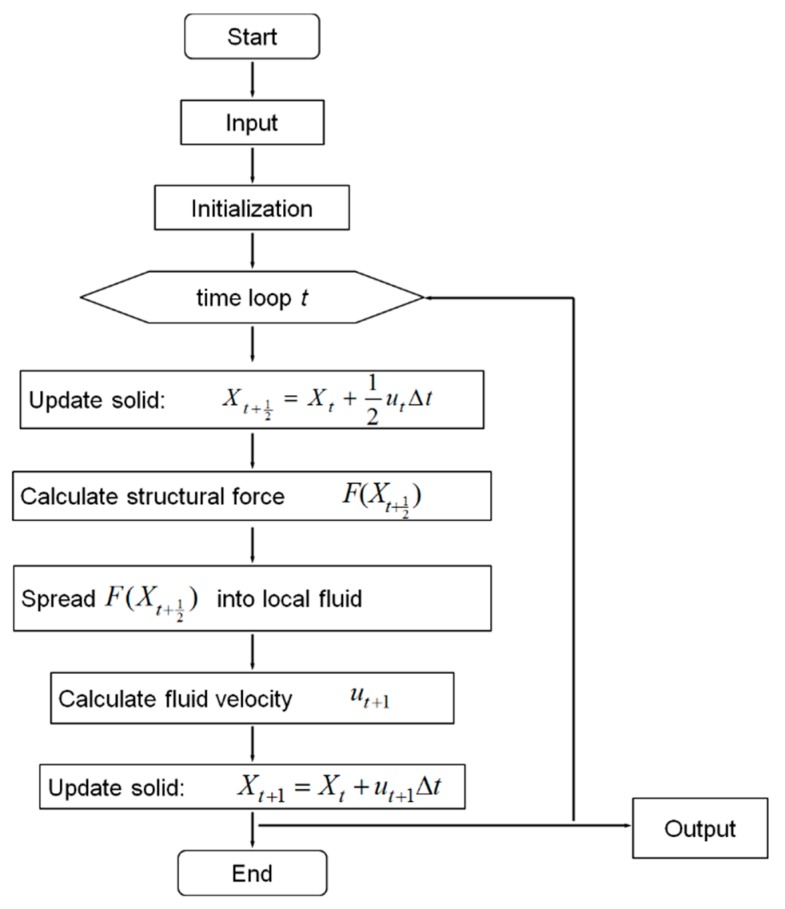
The algorithm flow chart of immersed boundary method.

The flow chart of the algorithm for the program is shown in Figure 2, which is a second order accurate Runge–Kutta method based primarily on the midpoint rule [25]. The fluid-structure interaction model was validated for a sphere settling process [67] and a lateral migration process [68] in a viscous fluid (Figures S1–S5). Validation studies for fluid-structure interaction, cell tumbling and tank treading motions (Figure S6, parameters listed in Table S1), as well as NP diffusion (Figures S7–S8) are shown in the Supplementary Materials, which is provided in the supplementary document.

## 3. Model Setup and Parametric Study

Research has shown that particles in the core region of the vessel migrate toward the cell-free layer regions, where the migration process can be modeled as diffusion [17,18]. This migration is influenced by physical conditions, such as hematocrit level (*H_t_*), cell membrane stiffness (*k_s_*), particle size (*r*), shear rate (η), fluid viscosity (*ν*) and cell size (*d_c_*). In this study, two parameters (hematocrit level and shear rate) are considered, while the other parameters are kept constant [13]. NP dispersion is first studied under pure shear flow conditions at different shear rates for a given hematocrit level. This pure shear flow setup was used to explore the NP dispersion rate when cells undergo tumbling and tank treading motion [69,70,71]. Then, the study is extended to investigate NP dispersion in channel flow at different hematocrit levels, which was used to model the blood flow in capillaries with a physiologically-relevant shear rate between 0 and 500 s^−1^ [72].

In the study, RBCs were assumed to be healthy with typical physical parameters and with a size of 8 μm. NPs were assumed to be spherical with a typical size of 100 nm. For simplicity, surface charges were neglected, so that NPs did not adhere to other NPs or to the RBCs. It is noted that the NP concentration was kept relatively low in order to more readily ascertain the effect of RBC motion on NP dispersion. Dynamic viscosity of the fluid was fixed at 1 × 10^−3^ Pa·s. Through dimensional analysis, an empirical function between diffusion coefficient, shear rate, cell size and hematocrit was defined as: $\frac{D}{d_{c}^{2}\eta }=f(H_{t})$, where *D* is the dispersion rate, *d_c_* is the cell size, *η* is the shear rate and $H_{t}$ is hematocrit. The same dimensionless dispersion rate was also suggested in [15]. This formula was validated through simulations presented in later sections.

**Figure 3:**
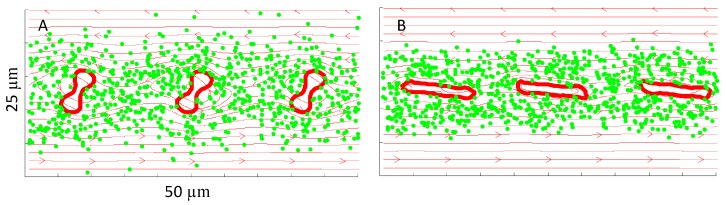
Interaction between nanoparticle (NP) and red blood cell (RBC) at shear rates of (**A**) 40 s^−1^ (RBC tumbling) and (**B**) 200 s^−1^ (RBC tank treading). The bold red lines outline the RBC membranes, while the green markers denote NPs. Flow streamlines are shown in the background.

The test case consisted of a rectangular fluid domain with a 50-µm length, a 25-µm width and a lattice grid size of 0.5 µm, as shown in Figure 3A. Simulation with a finer grid of 0.25 µm showed a similar fluid velocity field. In the shear flow case, the top and bottom surfaces were defined as velocity boundaries, while the left and right edges of the domain were modeled as periodic boundaries. In the channel flow case, a parabolic velocity profile was applied at the left inlet boundary, and the right outlet boundary was modeled as an open condition. Non-slip boundaries were defined along the upper and lower surfaces. Notice that the physical system has to be converted into LB units through reference dimensions, such as length (*dx*), time (*dt*) and mass (*dm*). Here, we select *dx* = 0.5 µm. The reference mass *dm* = ρ*dx*^3^ = 1.28 × 10^−16^ kg assuming the blood plasma density is 1025 kg/m^3^. Depending on the Reynolds number, a relaxation time τ is typically recommended between 0.8 and 1.0; see [73,74]. As suggested in [44], a relaxation time τ of 1.0 was used for all simulations for computational efficiency and larger time steps. The time step *dt* can be determined through normalizing the fluid viscosity $\frac{\tau -0.5}{3}=\nu \frac{dt}{dx^{2}}$, where ν is the kinematic viscosity of the fluid. Thus, the time step can be calculated as 4.2 × 10^−8^ s. Other parameters can be normalized, as well, since the basic reference values for length, time and mass have been selected. As all of the simulations performed here have low Reynolds numbers, the compressibility effect of the Mach number can be safely ignored.

RBC membranes were modeled as bi-concave curves with the dimensions shown in Figure 1A. A single RBC was composed of 52 nodes. The cell parameters were selected based on recommended values reported in the literature [53,75], as listed in Table 1. The artificial area constraint *k_a_* = 1 was selected so that the area change was within 1%. Periodic boundary conditions (PBC) were applied to the left and right boundaries of the fluid domain for both RBCs and NPs. The area ratio between RBCs and the fluid domain was defined as the hematocrit level.

**Table 1:** Cell membrane model parameters.

Parameters	Specified Value	Recommended Range	References
Stretching coefficient *k_s0_*	5 µN/m	5~12 µN/m	[53,75]
Bending coefficient *k_b_*	8 × 10^−19^ J	2 × 10^−19^ to 1 × 10^−17^ J	[53,75]

## 4. Results and Discussion

### 4.1. NP Dispersion under Pure Shear Flow

The NP dispersion rate was studied over a range of shear rates for a single layer of three cells. This setup was designed to eliminate the cell-cell interaction between different layers, so that we can focus on the shear rate effect on NP dispersions. Shear rates ranging from 20 to 500 s^−1^ were selected in order to cover both the RBC tumbling and RBC tank treading regions of the flow regime. For all shear rates investigated in the study, the dimensionless number *ηt* was held at 25. Therefore, the simulation time is longer for lower shear rate case. Three RBCs and 792 NPs were considered for each simulation. Snapshots of the interaction between NPs and RBCs at shear rates of 40 s^−1^ and 200 s^−1^, representative of RBC tumbling and RBC tank treading regions of the flow regime, respectively, are shown in Figure 3 (see the corresponding movies in the Supplementary Materials).

**Figure 4:**
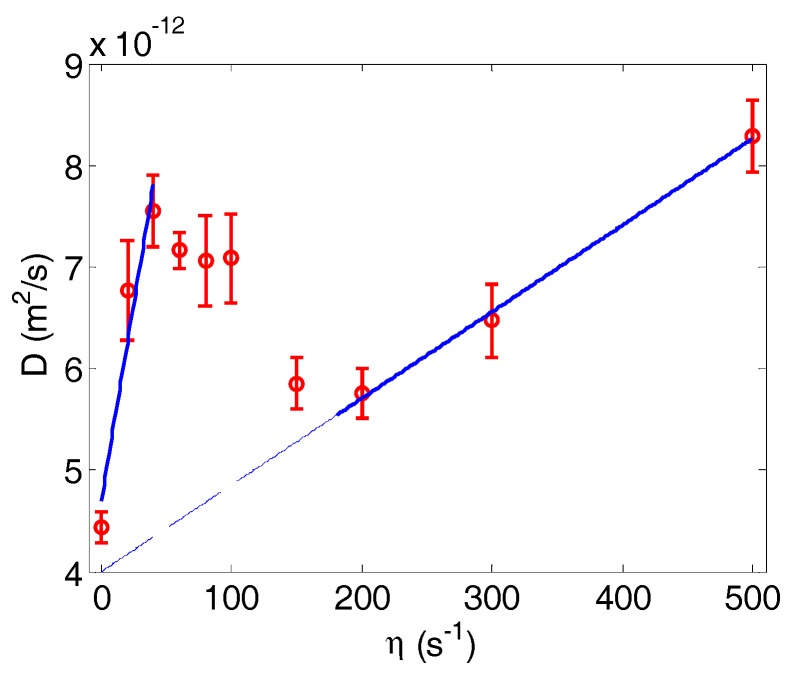
NP dispersion rate as a function of shear rate. Error bars indicate the standard variance for three simulations. RBCs undergo tumbling motion at a low shear rate (*η*< 40 s^−1^) and tank treading motion at a high shear rate (η > 200 s^−1^). In between, there is a transition region. Linear regression lines for the tumbling and tank treading regions are shown, as well.

The mean square displacement over the *y* direction at different shear rates was calculated to obtain the NP dispersion rates, as shown in Figure 4. It shows that the dispersion rate is strongly influenced by cell motion. In the RBC tumbling (η < 40 s^−1^) and RBC tank treading (η > 200 s^−1^) regions of the flow regime, the NP dispersion rate is approximately linear with the shear rate. Between 40 s^−1^ and 200 s^−1^, there is a region where RBC motion transits from tumbling to tank treading motion. In this transition region, there is a drop in NP dispersion with increased shear rate. This shows that the cell tumbles first and then it deforms to a shape that cannot sustain tumbling motion, then evolves into the tank treading shape. Thus, the overall average dispersion rate is decreased compared to the pure tumbling regime, which leads to a peak around a shear rate of 40 s^−1^. For the range of shear rates investigated in the study, the dispersion rate initially increases in the tumbling region, then decreases in the transition region and increases again with the shear rate in the tank treading region. A linear regression model was used to fit both the tumbling (first three data points at a low shear rate) and tank treading data (last three data points at a high shear rate),

$$\{\begin{array}{c} 7.8\times 10^{-14}\eta +4.7\times 10^{-12}\begin{array}{cc} & \text{tumbling } \end{array}\\ 8.5\times 10^{-15}\eta +4.0\times 10^{-12}\begin{array}{cc} & \text{tank treading} \end{array} \end{array}$$

where η is the shear rate. The formulae indicate that the effect of shear rate on NP dispersion in the tumbling region is roughly an order of magnitude larger than that in the tank treading region. This can be attributed to larger RBC motions in the tumbling region, where RBCs undergo rigid-like rotations that trigger larger local flow disturbances that promote the dispersion of adjacent NP away from the cell. It is also worth noting that the constant terms in the formulae are close to the NP thermal diffusion coefficient. The theoretical diffusion rate for 100-nm particles at a temperature of 300 K is about $4.4\times 10^{-12}$ m^2^/s. This observation agrees with the physical requirement that the dispersion rate should be close to thermal diffusion in the absence of shear flow. Therefore, for a given hematocrit level, and with η < 40 s^−1^ and η > 200 s^−1^, the dispersion rate *D* can be written as:

(16) $$D=k\eta +D_{0}$$

where $D_{0}$ is the thermal diffusion coefficient and *k* is a constant that depends on the hematocrit level. It is noted that this formula is readily adaptable to different particle sizes, because the constant term *D_0_* already contains the particle size effect. The contribution of RBC motion is represented in the constant k. It is noted that the influence of particle concentration on dispersion rate has been neglected.

### 4.2. NP Dispersion under Channel Flow

**Figure 5:**
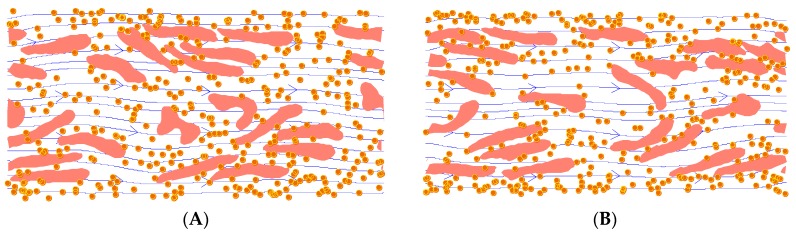
Snapshots from a channel flow simulation for a cell-particle mixture with a hematocrit level of 23.5% and a shear rate of 200 s^−1^ at 0.26 s (**A**) and 0.46 s (**B**). Fluid streamlines are shown in the background, while the yellow markers represent 100-nm nanoparticles. For illustration purposes, cells and particles are not shown to scale. A short movie of the simulation is provided in the Supplementary Materials.

It should be noted that the pure shear flow case does not exist in physiological flow. While the shear case provides an understanding of NP dispersion enhancement under different shear flow rates, it is important to simulate NP dispersion in realistic channel flow cases. Figure 5A,B presents snapshots of NP dispersion in a channel flow simulation with a hematocrit of 23.5% and a shear rate of 200 s^−1^ at 0.26 s and 0.46 s, respectively. The channel width is 25 µm. For these simulations, the specified shear rate was measured as the shear rate at the wall, unless noted otherwise. Due to the increased cell volume, compared to the pure shear flow simulations, the number of NPs was reduced to 378. The NPs were initially positioned in the core region of the channel. Since the shear rate is linearly changing across the channel, RBCs did not exhibit distinctive motions, such as tumbling or tank treading, as shown in the previous pure shear flow. The higher hematocrit and cell-cell interaction also confined the cell motion in the flow. The majority of the cells behaved like a tank treading motion, while some cells in the core region were bended or folded in the channel due to the symmetry of the velocity near the center line of the channel. The RBC motion under other hematocrit levels and shear rates was similar to Figure 5. However, they are not shown here.

**Figure 6:**
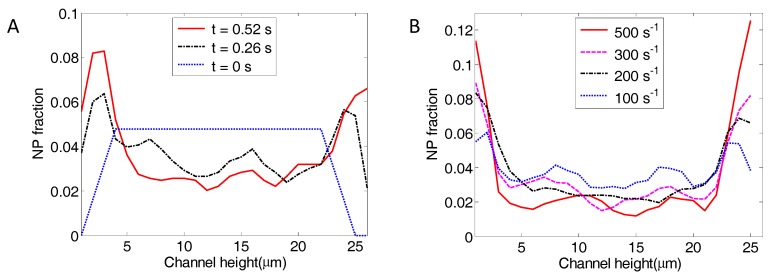
The NP fraction across the channel height for a hematocrit level of 23.5%. (**A**) NP fraction at *t* = 0, 0.26 s and 0.52 s for a shear rate of 200 s^−1^; and (**B**) NP fraction at *t* = 0.52 s for shear rates of 100 s^−1^, 200 s^−1^, 300 s^−1^ and 500 s^−1^.

As illustrated in Figure 5A,B, the NPs tend to migrate toward the wall. In order to characterize the NP distribution across the channel, the channel width was divided into bins of 1 µm. The number of NPs within each bin was counted and divided by the total number of NPs to obtain the NP fraction within each bin. The NP fraction across the channel height is plotted in Figure 6A at time points of 0, 0.26 and 0.52 s, for a characteristic shear rate of 200 s^−1^. Figure 6B presents the NP fraction across the channel height at *t* = 0.52 s under shear rates of 100 s^−1^, 200 s^−1^, 300 s^−1^ and 500 s^−1^. The NP fractional values shown in the figure are the average of three sample runs using different random seeds for the NP Brownian motion model. Figure 6B clearly demonstrates that particle migration speed toward the channel walls increases with shear rate.

**Figure 7:**
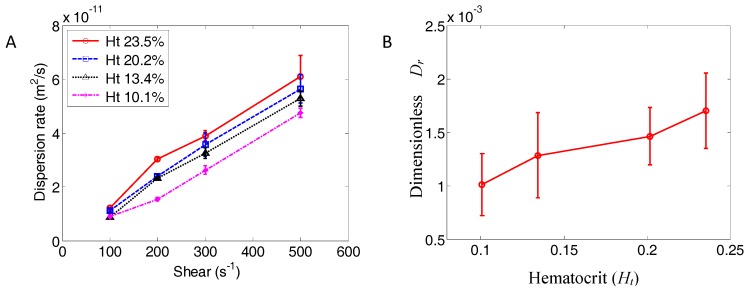
Dispersion rate of NPs in blood flow. (**A**) NP dispersion rate at different hematocrit (Ht) and shear rates; (**B**) relationship between dimensionless dispersion rate (*D_r_*) and hematocrit (*H_t_*). Error bars show the standard variance from three samples.

In order to characterize NP migration speed, the dispersion rate was calculated from the NP mean square displacement. The dispersion rates for different hematocrit levels, and at various shear rates, are shown in Figure 7A. From the pure shear simulation results shown in Equation (14), a modified dimensionless dispersion rate was developed,

(17) $$D_{r}=\frac{D-D_{0}}{d_{c}^{2}\eta }=f(H_{t})$$

The dimensionless dispersion rate $D_{r}$ is plotted in Figure 7B, where the error bars show standard variance for three sample runs.

Figure 7 shows that the lateral dispersion of NPs (*i.e.*, migration of NPs toward the vessel walls) is much larger than what is predicted by thermal diffusion alone. This migration is influenced by both the hematocrit level and the shear rate. While the relationship between dispersion rate and shear rate is approximately linear (Figure 7A), the relationship between dispersion and hematocrit is not fully linear (Figure 7B). Nevertheless, a best fit line with reasonable approximation can be written as:

(18) $$D_{r}=\frac{D-D_{0}}{d_{c}^{2}\eta }=4.643\times 10^{-3}H_{t}+5.834\;\times 10^{-4}$$

**Figure 8:**
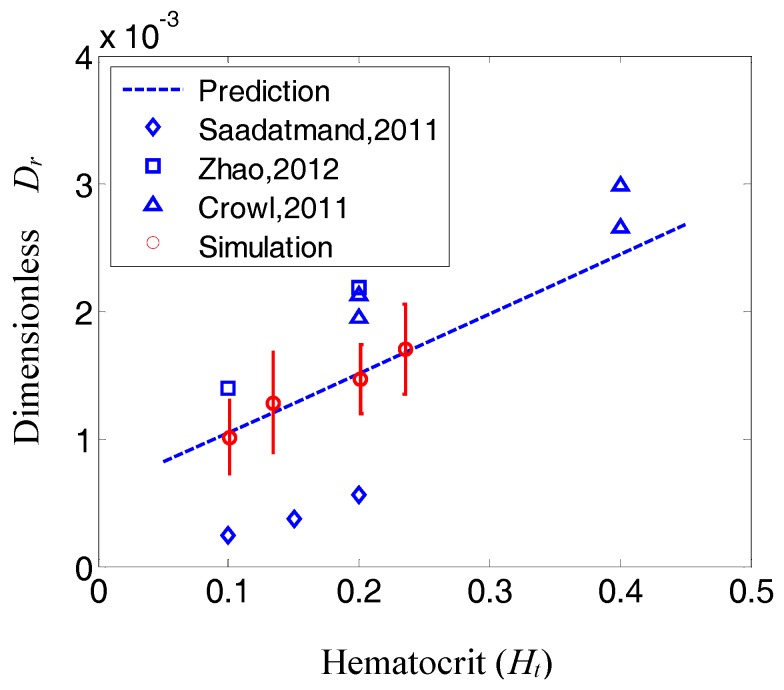
Comparison of the particle dispersion rate predicted from Equation (18) with the data reported in the literature. The dashed line is the prediction from Equation (18).

Particle dispersion rate predictions from Equation (18) were also compared to dispersion rates published in the literature for platelets [15,76] and 1-µm particles [13]. It is noted that the comparison of Equation (18) with platelets and microparticles was considered due to the lack of the NP dispersion rate in the literature. The predictions and measured dispersion rates are summarized in Table 2. The dimensionless dispersion rate is also plotted in Figure 8. As shown in both the table and the figure, the dispersion rate predictions using Equation (18) are in good agreement with measured rates reported in the literature. Discrepancies between the predicted and measured dispersion rates may be due to the linear correlation assumption for hematocrit and/or the effect of particle concentration, which was very low for this study and was assumed to have a negligible effect on NP dispersion. Another possible reason is that platelets generally have on average an aspect ratio of 1.4, and this may be a confounding factor and contribute to the differences [77,78]. Nevertheless, the order of magnitude of the dispersion rate, as well as its trend with hematocrit are correctly predicted by Equation (18).

**Table 2:** Comparison of particle dispersion rate predictions from Equation (18) with dispersion rates reported in the literature.

Ht	Shear (s^−1^)	Dispersion Rate (cm^2^/s)	Prediction (cm^2^/s)	Reference
[0.2, 0.4]	400	[0.5, 0.68] × 10^−6^	[0.39, 0.63] × 10^−6^	[76]
[0.2, 0.4]	1100	[1.5, 2.1] × 10^−6^	[1.1, 1.7] × 10^−6^	[76]
[0.1, 0.15, 0.2]	44.8	[8.2, 11.9, 17.2] × 10^−9^	[31.3, 37.9, 44.6] × 10^−9^	[13]
[0.1, 0.2]	804	[0.9, 1.4] × 10^−7^	[5.4, 7.8] × 10^−7^	[15]

## 5. Conclusion and Future Work

This paper presents a numerical study on NP dispersion in blood flow considering the influence of RBC motion and deformation. Analytical formulae, which did not exist from previous modeling results [3,16], were proposed to characterize NP migration to the vessel walls as a function of shear rate at different hematocrit levels. The formula’s predictions agree well with data reported in the literature, thereby facilitating practical modeling of NP transport and distribution in large-scale vascular systems.

In the future, the model will be extended to 3D to explore NP binding and distribution in capillary vessels. The proposed formula for the dispersion rate will also be used to evaluate NP transport and distribution in a large-scale vascular network.

## Supplementary Materials

Figures S1–S9, Table S1 and three short movies are available online at http://www.mdpi.com/2079-4991/6/2/30/s1.
