# Supplementary material for: Characterization of Nanoparticle Dispersion in Red Blood Cell Suspension by the Lattice Boltzmann-Immersed Boundary Method
Source: Nanomaterials (Basel). 2016 Feb 5;6(2):30. doi: 10.3390/nano6020030 (PMC5302481; doi:10.3390/nano6020030)
Supplement: Supplementary file 1 [file nanomaterials-06-00030-s001.zip › nanomaterials-06-00030 supplementary.pdf]

Supplementary Materials: Characterization of Nanoparticle Dispersion in Red Blood Cell Suspension by the Lattice Boltzmann-Immersed Boundary Method

Jifu Tan, Wesley Keller, Salman Sohrabi, Jie Yang and Yaling Liu

1. Fluid Solver Benchmark

To test the fluid solver, we created a 2D channel flow case with 20 × 20 lattices over the length and width. A pressure gradient (or force density) of 1.47×10^{-4} in dimensionless units was applied to the lattice Boltzmann algorithm. The relaxation parameter tau = 1, so the viscosity is 1/6; while the outlet is set to open so that velocity gradient along the flow direction is zero. The top and bottom boundaries were assumed to be nonslip using the bounce back algorithm.

From the analytical solution,

$$V_{max} = \frac{R^2}{4u}\frac{\partial p}{\partial z} \tag{1}$$

We know that the maximum fluid velocity of the parabolic profile is $V_{\max} = 0.02$. We ran the simulation to Time Step 1200, where the fluid velocity already reached the steady state. The fluid velocity across the channel at the middle of the channel length was selected and compared to the theoretical parabolic profile. The comparison between the simulation and the analytical solution is shown in Figure S1.

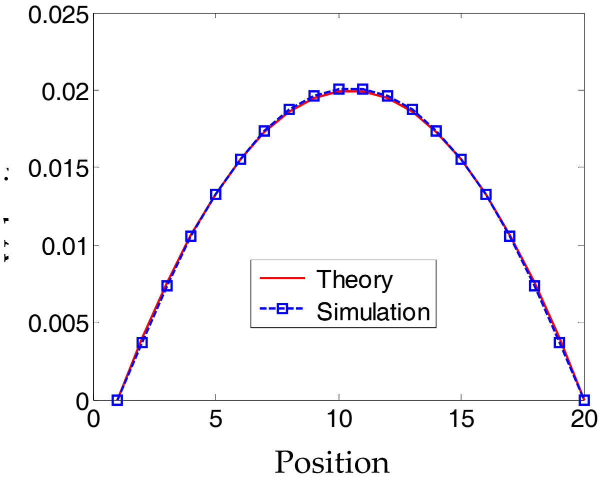

Figure S1. The comparison of the parabolic velocity profile from lattice Boltzmann (LB) simulation and the theoretical values.

As shown from the figure, the numerical simulation data agree well with the theoretical predictions. The relative error of the maximum velocity is 0.4%. Thus, the code can correctly solve the fluid flow.

2. Fluid Structure Interaction Benchmark: Particle Settling and Lateral Migration

2.1. Particle Settling

Sphere settling in a viscous fluid is widely used as a benchmark for fluid structure interaction (FSI) simulation [1]. The process involves placing a solid sphere in a static fluid and allowing it to accelerate downward under gravity loading until it reaches a steady velocity, where the resultant drag force balances the gravity load. In our FSI benchmark, the sphere is modeled as a rigid 2D ring structure.

The motion of the ring is interpolated from the local fluid velocity. A rigid boundary, however, is very difficult to achieve in the immersed boundary method. Therefore, the modeling approach developed by Fogelson [2] and Feng [3] was adopted. The model produces an effectively rigid particle surface using stiff elastic fibers. This is accomplished by using virtual images of the surface nodes undergoing rigid motion. A restorative force is applied to the nodes that deviate from the position of the virtual image. Additional details for the approach can be found in the referenced studies.

The fluid channel for the benchmark simulation was 4 cm in length and 1 cm in width. The sphere had a diameter of 0.1 cm and was placed in the fluid at approximately 0.8 cm away from the top, along the channel center line. The density of the sphere was 1001 kg/m^3. The restorative stiffness used for the effective rigid boundary was 1 × 10^{-4} N/m. The fluid was taken as water with a density of 1000 kg/m^3 and a viscosity of 1 × 10^{-3} Pa·s. The lattice size *dx* was 1 × 10^{-4} m, and the time step *dt* and lattice Boltzmann relaxation parameter τ were 1.667 × 10^{-3} s and 1.0, respectively.

Following [4], the theoretical terminal velocity of a cylinder (2D) is $V_s = \sqrt{\dfrac{\pi g D(\rho_s - \rho_f)}{2 C_d \mu \rho_f}}$, where the drag coefficient $C_d = \dfrac{8\pi}{\mathrm{R_e} \log\left(\dfrac{7.4}{\mathrm{R_e}}\right)}$, where *D* is the diameter, μ is viscosity, ρ is the density and *s* and *f* stand for solid and fluid, respectively. In order to convert from a node-connected ring to a disk, an associated nodal tributary area had to be defined, so that the distributed gravitational force could be treated as an equivalent nodal force system.

For this study, the associated tributary area was approximated as $dA = \left(\dfrac{\pi D}{n_s}\right)^2$, where n_s is the total number of nodes.

The predicted terminal velocity from the simulation was 4.6 × 10^{-4} m/s, which compares very well with the theoretical Stokes formula prediction of 4.42 × 10^{-4} m/s, as shown in Figure S2. The difference between the theoretical and simulation-based terminal velocity prediction is within 3.8%, which indicates that the FSI code correctly reproduces the kinematics of the sphere in a viscous fluid.

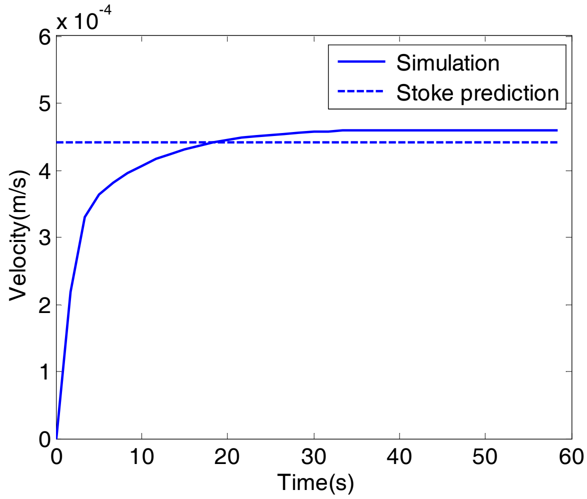

Figure S2. Particle settling velocity in simulation compared to theoretical terminal velocity.

2.2. Lateral Migration

We decided to benchmark particle lateral migration with the data reported in Feng *et al.* [5]. The key idea behind the immersed boundary method is to update the solid position with local fluid velocity and feedback the fluid with a force term coming from the defamation of the solid. Therefore, it is ideal for soft structures, but not a good choice for a rigid solid. Here, we modeled a rigid cylindrical particle using a relatively larger stiffness and smaller time steps. The cylindrical particle

only has peripheral nodes, like a ring. We select the lattice Boltzmann reference value as $dx = 1 \times 10^{-4}$ m, $dt = 9.067 \times 10^{-5}$ s, tau = 0.5272. The fluid channel has 80 × 200 grids with nonslip top and bottom surfaces. The inlet and outlet boundaries are set to be periodic. The solid spring constant is 5×10^{-3} N/m, and the bending stiffness is 8×10^{-17} Nm. We set the density difference as 7.6×10^{-4} (assuming fluid density is one), so that the Re = 1.03, which is consistent with the reported Re. Other conditions are set exactly the same as [5]. Two snap shots of the streamline and the deformation of the cylindrical particle are shown in Figure S3. Since the ring is not perfectly rigid, it shows some deformation during the migration process, particularly when it is close to the wall.

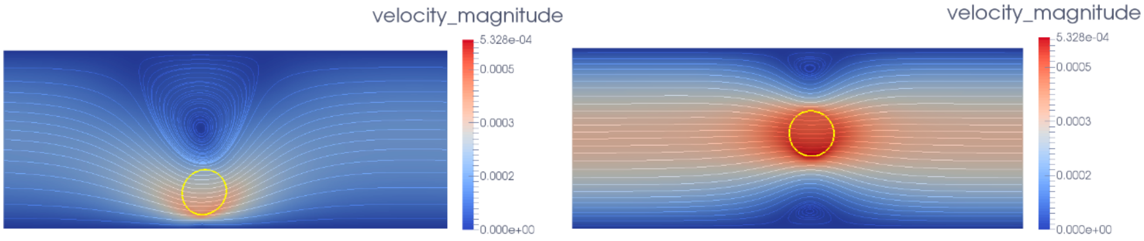

Figure S3. Snapshots of the particle settling modeling at Time Steps 300,000 (**left**) and 4,800,000 (**right**). Since the ring is not perfectly rigid, it shows some deformation at a large shear rate close to the wall.

The comparison of the particle trajectories between our model and the data from [5] is shown in Figure S4. It shows that the overall trend and the migration process predicted from our model are consistent with the data from [5]. However, in our model, the ring travels about a five-times greater distance along the fluid channel. This is likely due to the fundamental difference between a ring and disk. For example, we calculate the ratio between the area of a disk and the perimeter of a ring, $\pi r^2/(2\pi r) = r/2 = 10/2 = 5$, as the diameter of the ring is $L/4 = 20 = 2r$. The soft ring may also lead to the difference between our model prediction and the reference data. However, the model can give qualitatively correct prediction of the particle lateral migration.

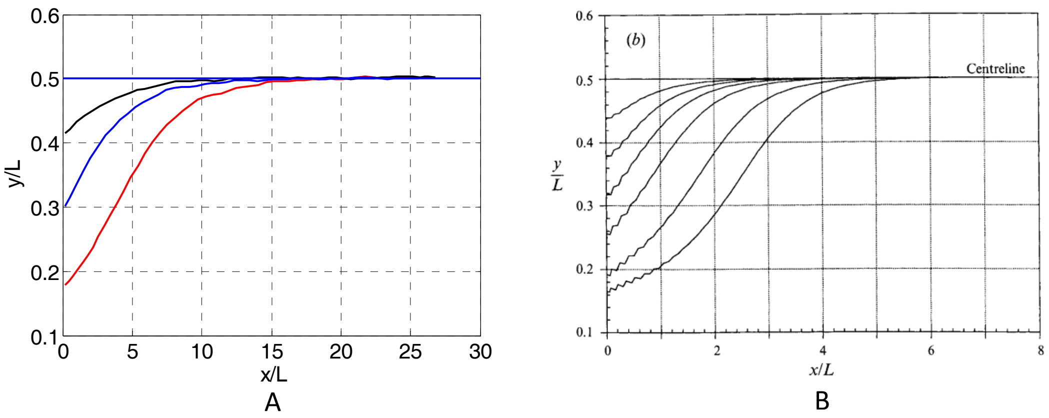

Figure S4. Settling trajectories for particle released from different initial positions. Re = 1.03, channel width $L = 4d$, d being the circular particle diameter. (**A**) Our simulation data. (**B**) Published literature data obtained from Feng *et al.* Reproduced with permission of [5]. Copyright Cambridge University Press, 1994.

We also performed a particle migration benchmark with different Re numbers. We set the density difference as 0.00238 and 0.00615 (assuming fluid density is one), so that the Re = 3.23 and 8.33, which is consistent with the reported Re. Other conditions are set exactly the same as [5].The trajectory of the particles is shown in Figure S5.

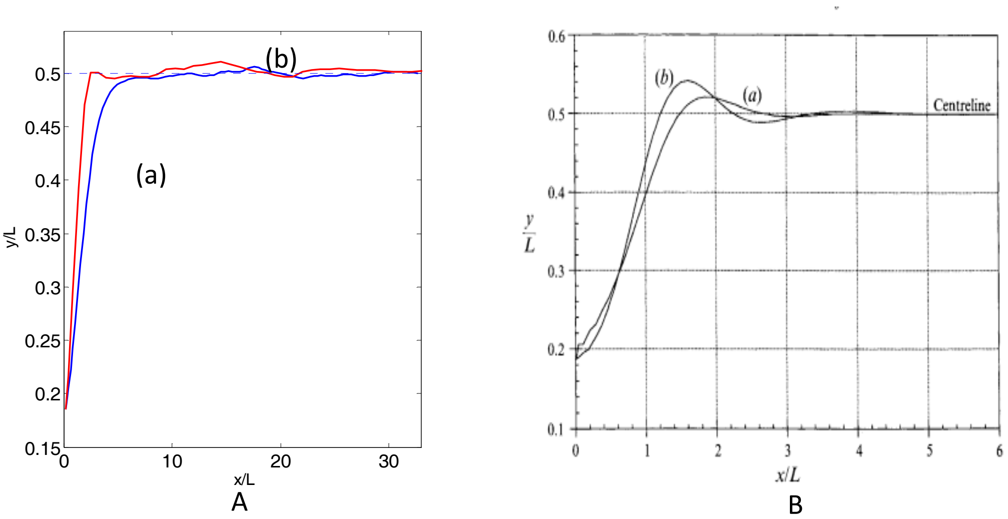

Figure S5. The particle setting trajectory at (a) Re = 3.23, (b) Re = 8.33. (**A**) Our simulation results; (**B**) published literature data obtained from Feng *et al.* Reproduced with permission of [5]. Copyright Cambridge University Press, 1994.

As shown in the figure, we can see the slightly overshoot trajectory of the particle before the particle reaches equilibrium. The overshoot phenomena is induced by the particle inertia. In the immersed boundary method, the particle is assumed to always follow the local fluid velocity. Thus, it is difficult to create exactly the same trajectory overshoot as shown in [5]. In our particle dispersion study, the inertia effect is not important, as the Re for our flow case is very low. For example, with a channel width of 25 μm, the inlet velocity is 6.25×10^{-3} m/s for the highest shear rate of 1000 s^{-1} studied in our simulation. The corresponding Re number based on cell diameter is 0.05, which is very low. Thus, we believe that we can safely ignore this transient effect induced by the inertia.

However, it should be noted that the lateral migration of particles at a high Re number can be modeled if a different coupling approach is used. For example, Tony Ladd [6,7] treated the settling solid as a moving boundary for the fluid domain, and then, the hydrodynamic force was applied back to the solid to determine its motion. A similar approach was also used in a particulate suspension study [8,9]. This approach is more general and suitable for rigid particles.

3. Cell Behavior under Shear

It is well known that red blood cells and droplets will undergo tumbling or tank treading motion under shear, depending on the shear rate and membrane stiffness. Capillary number is typically used to characterize the cell motion. It is defined as $Ca = v_0 \eta r / k_s$, where v_0 is the reference viscosity, η is the shear rate, r is the cell radius and k_s is the stretching resistance. The cell parameters for the simulations are shown in the Table S1. The fluid domain was 20 μm by 20 μm. The fluid was taken as water with a density of 1000 kg/m^3 and a viscosity of 1×10^{-3} Pa·s. The lattice size *dx* was 5×10^{-7} m, and the time step *dt* and the relaxation parameter τ were 4.2×10^{-8} s and 1.0, respectively. The typical cell diameter was 8 μm modeled with 52 nodes.

Table S1. Parameters for the cell model used in the study.

Parameters	Value Used	Suggested Value	References
Spring constant k_{s0}	5 μN/m	5–12 μN/m	[10,11]
Bending constant k_b	8×10^{-19} Nm	2×10^{-19}–1×10^{-17} Nm	[10,11]

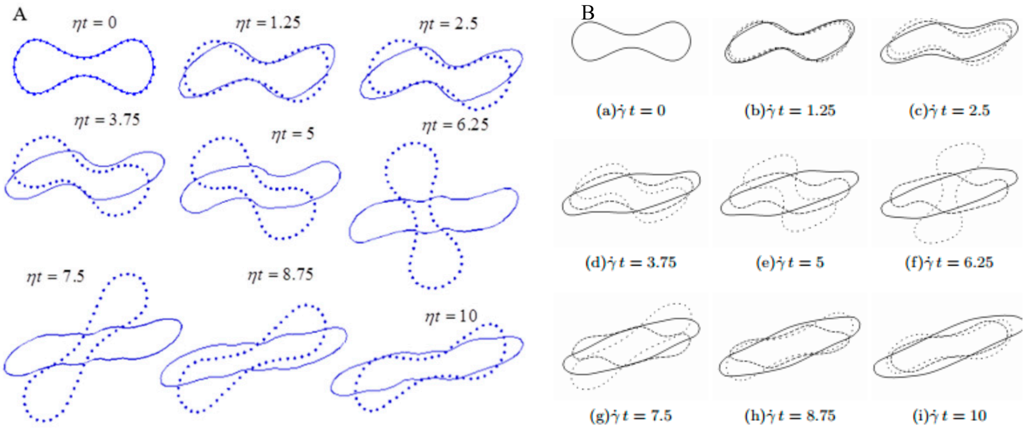

Figure S6. (**A**) Snapshots of tumbling and tank treading motions of red blood cell in shear flow from our model. ηt is the product of the shear rate and time, a dimensionless number. The solid lines represent cell profiles for a shear rate of 200/s, while the dashed lines represent cell profiles for a shear rate of 20/s. (**B**) Data obtained from [12]. (Reproduced with permission of [12]. Copyright Royal Society of Chemistry, 2013.

In these simulations, tank treading motion occurs at $C_a = 0.16$, while tumbling motion occurs at $C_a = 0.016$. These observations are consistent with the physical requirement that tumbling motion occurs at a low capillary number and tank treading motion occurs at a high capillary number. In [12], the tank treading case (solid line) is performed at $C_a = 0.5$, and the tumbling case (loose dashed line) is performed at $C_a = 0.1$. Notice that the membrane energy used in [12] is a 3D model based on a continuum approach, which is different from our model here. In their calculation, they used the shear modulus of the membrane, and in our case, we used the spring constant. However, the shape of the cell and its inclined angle are qualitatively similar. We also compare the inclined angle of the cell under tank treading motion. The cell inclined angle ϕ is defined as the angle between the cell's longest axis and the flow direction and can be used to benchmark cell dynamics under shear. The cell inclined angle ϕ at $C_a = 0.16$ was $\phi/\pi = 0.07$, which is within the range of 0.085 ± 0.03 reported in [13]. Therefore, the above quantitative results validate our FSI code for modeling cell tumbling and tank treading motion.

4. Nanoparticle Diffusion

Brownian motion was used to capture the nanoparticle motion. The thermal noise is modeled through a random force term $\mathbf{F}_r$ that satisfies,

$$\langle \mathbf{F}_r(t) \rangle = 0 \tag{2}$$

$$\langle \mathbf{F}_r(t)\mathbf{F}_r(t') \rangle = 2k_B T \varsigma \delta(t-t')\mathbf{I} \tag{3}$$

where k_BT is thermal energy, ς is the friction coefficient, $\delta(t-t')$ is the Dirac delta function and $\mathbf{I}$ is the unit-second order tensor.

Without any fluid flow, the random force will excite nanoparticles to walk randomly through the fluid domain. The simulated diffusion coefficient can be calculated from the mean square displacement. It should be close to the thermal diffusion coefficient calculated by Einstein's formula. In our model settings, the fluid domain was 25 μm by 50 μm. The fluid was taken as water with a density of 1000 kg/m^3 and a viscosity of 1×10^{-3} Pa·s. The lattice size dx was 5×10^{-7} m; time step dt and the relaxation parameter τ were 4.2×10^{-8} s and 1.0, respectively. Three hundred seventy eight nanoparticles of a size of 100 nm were randomly positioned in the fluid domain. The temperature was set at 300 K.

The mean square displacement time history is plotted in Figure S7. The diffusion coefficient given by the half of the slope is 4.317 × 10^{-12} m^2/s, which is very close to the value given by Einstein's formula 4.39 × 10^{-12} m^2/s. Therefore, it shows that our code can reproduce the thermal diffusion of nanoparticles.

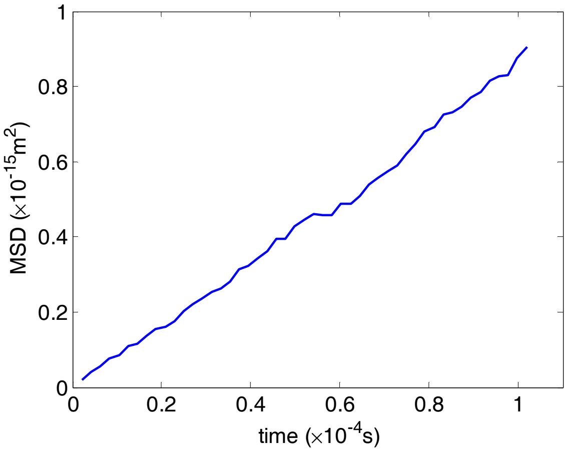

Figure S7. Mean square displacement (MSD) time history plot in the simulation in the absence of flow.

In our model, the fluid model does not have any thermal fluctuation. We plot out the mean kinetic energy of all of the nanoparticles and compared to the thermal energy, as shown in Figure S8. It shows that the kinetic energy fluctuates at different time steps. The time averaged ke/kBT following [14] is also plotted in the figure, as shown as the dashed line. It approaches one as more sampling points are used.

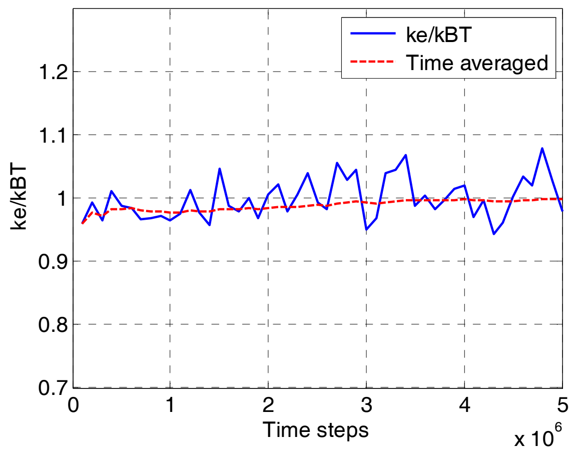

Figure S8. The ratio of particle kinetic energy to thermal energy at different time steps.

5. Potential Extension to 3D Modeling

The approach presented in this paper is readily extended into the 3D case. For example, we have a 3D case with less lattices (50 × 25 × 25) and 31 cells; it takes 24.3 h to model blood flow of 8.3 × 10^{-3} s, as shown in Figure S9. If we want to model the system in a few seconds, it would take thousands of hours. That is the reason we limited our study to 2D currently. One solution to this problem is to run it in parallel. Currently, we are still working on parallelization of the code.

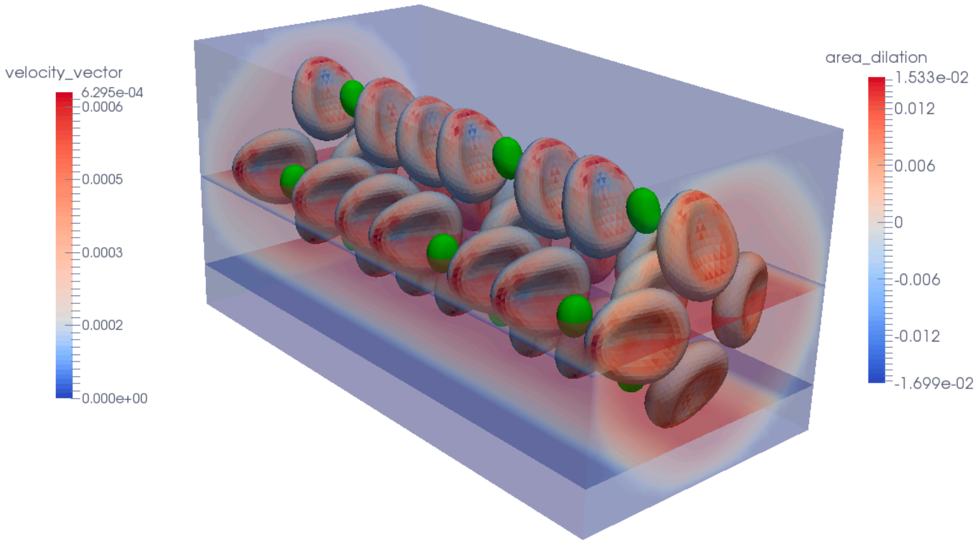

Figure S9. A preliminary 3D simulation of blood flow using the lattice Boltzmann-immersed boundary method. The fluid domain is 25 × 12.5 × 12.5 μm.

References

1. Zhang, L.; Gerstenberger, A.; Wang, X.; Liu, W.K. Immersed finite element method. *Comput. Methods Appl. Mech. Eng.* **2004**, *193*, 2051–2067.
2. Fogelson, A.L.; Peskin, C.S. A fast numerical method for solving the three-dimensional stokes' equations in the presence of suspended particles. *J. Comput. Phys.* **1988**, *79*, 50–69.
3. Feng, Z.-G.; Michaelides, E.E. The immersed boundary-lattice Boltzmann method for solving fluid–particles interaction problems. *J. Comput. Phys.* **2004**, *195*, 602–628.
4. Ghosh, S.; Stockie, J.M. Numerical simulations of particle sedimentation using the immersed boundary method. *Commun. Comput. Phys.* **2015**, *18*, 380–416.
5. Feng, J.; Hu, H.H.; Joseph, D.D. Direct Simulation of Initial-Value Problems for the Motion of Solid Bodies in a Newtonian Fluid. 1. Sedimentation. *J. Fluid Mech.* **1994**, *261*, 95–134.
6. Ladd, A.J.C. Numerical Simulations of Particulate Suspensions via a Discretized Boltzmann-Equation. 1. Theoretical Foundation. *J. Fluid Mech.* **1994**, *271*, 285–309.
7. Ladd, A.J. Numerical simulations of particulate suspensions via a discretized Boltzmann equation. Part 2. Numerical results. *J. Fluid Mech.* **1994**, *271*, 311–339.
8. Aidun, C.K.; Lu, Y.N.; Ding, E.J. Direct analysis of particulate suspensions with inertia using the discrete Boltzmann equation. *J. Fluid Mech.* **1998**, *373*, 287–311.
9. Basagaoglu, H.; Allwein, S.; Succi, S.; Dixon, H.; Carrola, J.T., Jr.; Stothoff, S. Two- and three-dimensional lattice Boltzmann simulations of particle migration in microchannels. *Microfluid. Nanofluid.* **2013**, *15*, 785–796.
10. Fedosov, D.A. Multiscale modeling of Blood flow and soft matter. In *Applied Mathematics*; Brown University: Providence, RI, USA, 2010.
11. Dao, M.; Li, J.; Suresh, S. Molecularly based analysis of deformation of spectrin network and human erythrocyte. *Mater. Sci. Eng. C* **2006**, *26*, 1232–1244.
12. Kruger, T.; Gross, M.; Raabe, D.; Varnik, F. Crossover from tumbling to tank-treading-like motion in dense simulated suspensions of red blood cells. *Soft Matter* **2013**, *9*, 9008–9015.
13. Zhao, H.; Shaqfeh, E.S.G.; Narsimhan, V. Shear-induced particle migration and margination in a cellular suspension. *Phys. Fluids* **2012**, *24*, doi:10.1063/1.3677935.
14. Basagaoglu, H.; Melchionna, S.; Succi, S.; Yakhot, V. Fluctuation-dissipation relation from a FLB-BGK model. *EPL* **2012**, *99*, 64001–64006.

© 2016 by the authors; licensee MDPI, Basel, Switzerland. This article is an open access article distributed under the terms and conditions of the Creative Commons by Attribution (CC-BY) license (http://creativecommons.org/licenses/by/4.0/).
